# Stopping in (e)motion: Reactive action inhibition when facing valence-independent emotional stimuli

**DOI:** 10.3389/fnbeh.2022.998714

**Published:** 2022-09-30

**Authors:** Simone Battaglia, Pasquale Cardellicchio, Chiara Di Fazio, Claudio Nazzi, Alessio Fracasso, Sara Borgomaneri

**Affiliations:** ^1^Department of Psychology, Center for Studies and Research in Cognitive Neuroscience, University of Bologna, Bologna, Italy; ^2^Department of Psychology, University of Turin, Turin, Italy; ^3^IIT@UniFe Center for Translational Neurophysiology, Istituto Italiano di Tecnologia, Ferrara, Italy; ^4^Institute of Neuroscience and Psychology, University of Glasgow, Glasgow, United Kingdom; ^5^Istituto di Ricovero e Cura a Carattere Scientifico (IRCCS), Fondazione Santa Lucia, Rome, Italy

**Keywords:** action inhibition, emotions, emotional body expression, stop-signal task (SST), valence-arousal

## Abstract

Emotions are able to impact our ability to control our behaviors. However, it is not clear whether emotions play a detrimental or an advantageous effect on action control and whether the valence of the emotional stimuli differently affects such motor abilities. One way to measure reactive inhibitory control is the stop-signal task (SST), which estimates the ability to cancel outright a response to the presentation of a stop signal by means of the stop signal reaction times (SSRT). Impaired as well as facilitated action control has been found when faced with emotional stimuli such as stop signals in SSTs and mixed results were observed for positive versus negative stimuli. Here, we aimed to investigate these unresolved issues more deeply. Action control capabilities were tested in 60 participants by means of a SST, in which the stop signals were represented by a fearful and a happy body posture together with their neutral counterpart. Results showed that both positive and negative body postures enhanced the ability to suppress an ongoing action compared to neutral body postures. These results demonstrate that emotional valence-independent emotional stimuli facilitate action control and suggest that emotional stimuli may trigger increased sensory representation and/or attentional processing that may have promote stop-signal processing and hence improved inhibitory performance.

## Introduction

Emotions play an essential role in our life, as they motivate action tendencies in response to the environmental changes and trigger adaptive behaviors to attain changeable goals. Nevertheless, empirical evidence suggests that emotions impact a variety of cognitive abilities, including executive functioning. One well-characterized executive function is response inhibition, which represents an important component and underlies behavioral flexibility by allowing us to stop highly automated, yet contextually inappropriate, actions (Bari and Robbins, 2013). When several cues in the environment compete for processing resources, emotional stimuli might receive prioritization over neutral stimuli and therefore affect cognitive processes (Kalanthroff et al., 2013) and thus our ability to proficiently control our actions. The ability to control prepotent responses can be investigated experimentally using a stop-signal task (SST), designed to provide a sensitive measure of the time taken by the brain to inhibit or suppress inappropriate reactive motor responses (Vince, 1948; Lappin and Eriksen, 1966; Logan et al., 1984; Borgomaneri et al., 2020a). SST requires participants to respond to a go stimulus and to abort the ongoing response when a stop signal is presented. The SST provides a measure of reactive control, by measuring stop signal reaction time (SSRT), which allows to estimate the time taken by the brain to inhibit or suppress inappropriate reactive motor responses, exploiting the race model (Logan et al., 1984). Several studies have used the SST to investigate how emotional stimuli are able to impact our ability to suppress inappropriate actions (Battaglia et al., 2021). Pessoa (2009) suggested that the impact of emotional experiences on behavior crucially depends on the threat level, which will determine if emotional content enhances or impairs behavioral performance. Indeed, the same authors (Pessoa et al., 2012) also demonstrated that emotions can either facilitate or reduce cognitive performance, likely as a function of the emotional potency of the stimuli involved: low-level emotional stimuli are able to enhance sensory representation and/or attentional processing, thus facilitating stop-signal processing and hence improving inhibitory performance, while high-level emotional stimuli seem to consume the processing resources needed for successful inhibitory performance and thus reduce action control. Although several subsequent studies have attempted to shed light on the complex interplay between emotional stimuli and action control, the results are still contradictory (Battaglia et al., 2021) and the factors able to modulate such diverse findings are still to be disclosed. A potential limitation of previous studies is that they have mostly investigated the ability of the negative versus neutral stimuli to impact on action control [see for a review Battaglia et al. (2021)]. However, negative and neutral stimuli differ not only in valence but also in the arousal level. Therefore, it is not possible to exclude that prolonged stopping latencies could be due to the arousal difference between negative and neutral information [see Pessoa et al. (2012)]. Few studies have employed positive emotions in SST (Verbruggen and De Houwer, 2007; Pessoa et al., 2012; Rebetez et al., 2015; Nayak et al., 2019; Williams et al., 2020; Gupta and Singh, 2021) and, interestingly, only Rebetez et al. (2015) and Gupta and Singh (2021) found different effects depending on the emotional valence of the go stimuli (i.e., stronger and lower interference for negative compared to positive stimuli, respectively) when the emotion was not relevant to the go task. In contrast, Williams et al. (2020) found no difference when the emotion was not relevant to the task, while a facilitation effect for positive versus negative stimuli emerged when the go task involved emotion discrimination. In partial agreement with these findings, Nayak et al. (2019) found shorter SSRT for positive versus neutral stimuli but not versus negative, when emotional stimuli were task-relevant. From these findings, it is clear that the question of whether or not the valence has an impact on action control is still to be resolved. In our recent study (Battaglia et al., 2022a), we demonstrated that very different kinds of negative stimuli (i.e., intrinsically negative stimuli such as fearful facial expressions or body postures, or fear conditioned stimuli such as the image of the SARS-CoV-2) have precisely the same ability to enhance our action control capabilities when task-irrelevant. However, in our previous studies we did not employ any control for valence, such as a positive stimulus. Here, we aimed to add this additional control by testing two different groups of participants in an online SST task using negative as well as arousal-matched positive emotional body postures as stop stimuli to test their ability to influence our action control with respect to their neutral counterpart.

## Methods

### Participants

A total of 90 right-handed healthy individuals were enrolled in the present study, 30 of whom took part in the pilot study to validate the visual stimuli, while the remaining 60 were involved in the main experiment involving the SST. All subjects had normal or corrected-to-normal visual acuity and declared they had no history of neurological or psychiatric disease; none of the participants was regularly taking any medication affecting the central or peripheral nervous system. Participants in the main experiment were randomly assigned to two groups: 30 to the Fear-Body group and 30 to the Joy-Body group. The number of participants was determined based on a power analysis, which indicated that a sample size of 30 participants is necessary to achieve a statistical power (1-β) of 0.99 (two-tailed α = 0.01; effect size *f* = 0.4) (Pessoa et al., 2012; Battaglia et al., 2022a); number of measurements = 2; correlation = 0.5, analysis performed with G*Power software (Faul et al., 2007). The groups were matched for age [*F*_(1,58)_ = 0.534; *p* = 0.47; η_*p*_^2^ = 0.009], years of education [*F*_(1,58)_ = 0.577; *p* = 0.45; η_*p*_^2^ = 0.009], and gender [χ^2^ (2, *N* = 60) = 0.635; *p* = 0.42]. Based on previous findings (Pessoa et al., 2012; Legrand and Price, 2020; Battaglia et al., 2021), which revealed an influence of psychological or psychiatric conditions (i.e., anxiety, depression, and impulsivity) on SST performance, we also investigated different personality states of the participants. Subjective levels of anxiety were measured through the State-Trait Anxiety Inventory (STAI; Trait-scale-Y2) (Spielberger et al., 1983), while subjective levels of impulsivity were measured using the Barratt Impulsiveness Scale-11 (BIS-11) (Patton et al., 1995). The STAI-Y2 consists of a 20-item self-report questionnaire providing an assessment of anxiety and evaluates how often respondents experience anxiety. The BIS-11 is a self-report questionnaire for the assessment of impulsiveness and is composed of 30 items assessing common impulsive or non-impulsive behaviors. Finally, the Hospital Anxiety and Depression Scale (HADS) (Zigmond et al., 1983) was administered to exclude clinically significant anxiety and depressive symptoms in our sample. The HADS is a 14-item questionnaire designed to assess levels of anxiety and depression that a person is experiencing, and consists of seven questions for anxiety and seven for depression. The two groups did not show any significant difference in terms of anxiety [STAI-Y2: *F*_(1,58)_ = 0.719, *p* = 0.40, η_*p*_^2^ = 0.0122; HADS-anxiety: *F*_(1,58)_ = 2.991, *p* = 0.09, η_*p*_^2^ = 0.049], HADS-depression [*F*_(1,58)_ = 2.48, *p* = 0.12, η_*p*_^2^ = 0.041], and BIS-impulsivity [*F*_(1,58)_ = 0.452, *p* = 0.50, η_*p*_^2^ = 0.007] scores.

Data collection was anonymous, and all participants gave their informed consent electronically through our online platform before the task. Data were hosted and stored on a private server and were password protected and accessible only by the corresponding authors. The study was conducted in accordance with the ethical principles of the World Medical Association Declaration of Helsinki and was approved by the Ethics Committee of the Department of Psychology of the University of Bologna.

### Experimental procedure

Participants performed an online web-version of the classical SST previously used in another recent study by our team (Battaglia et al., 2022a). The task was developed in-house using the jsPsych library version 6.1.0 (De Leeuw, 2015), based on JavaScript ES6,^1^ of a classical custom-made SST running local-only.

**FIGURE 1:**
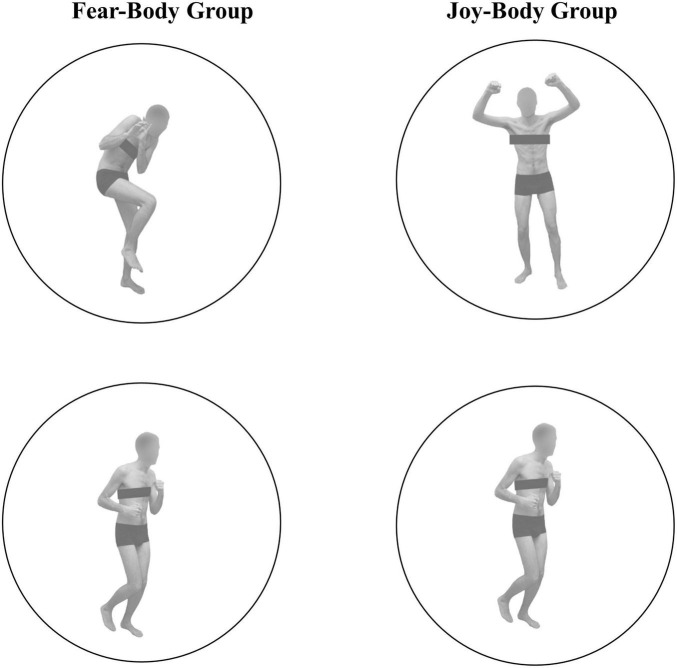
Visual stimuli used as Stop-Signal stimuli. In the Fear-Body group, stimuli consisted of two different body pictures with fearful and neutral body expression, and in the Joy-Body group stimuli were represented by happy and neutral body postures, previously used and validated in Borgomaneri et al., 2012, 2015a,b,c, 2017, 2020b, 2021.

In the present SST, Go stimuli consisted in the presentation of a black arrow pointing left or right, while two different body pictures (i.e., fearful/joyful and neutral expression) previously used in Borgomaneri et al., 2012, 2015a,b,c, 2017, 2020b, 2021 were used as Stop-signals (see Figure 1). Stimuli were edited to have the same shape, surface, complexity, colors, and contrast ratio with Blender (Blender Foundation, Amsterdam, Netherlands), and Adobe Photoshop CS6 software (Adobe, San Jose, California, USA).

**FIGURE 2:**
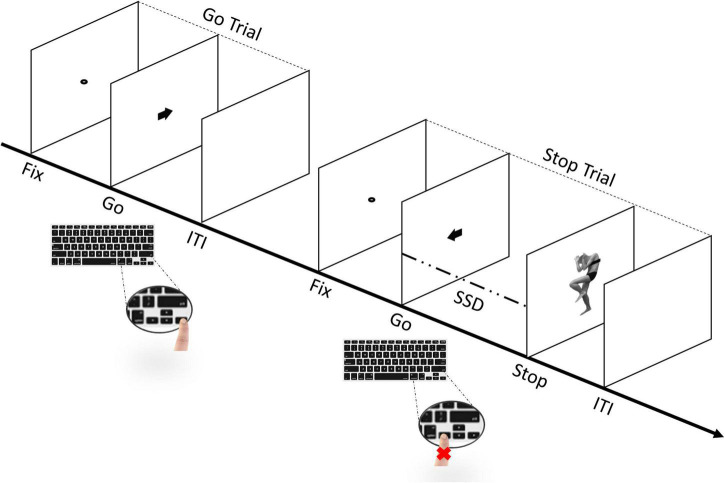
Sequence of trials in the stop-signal task (SST). The experimental task includes both go- and stop-trials (Lappin and Eriksen, 1966; Logan and Cowan, 1984b; Logan et al., 2014; Verbruggen et al., 2019). Participants perform a short practice block and, immediately afterward, four experimental blocks. Each block includes a total of 128 trials, of which 96 are go-trials (75%) and 32 are stop-trials (25%). In go-trials, participants respond to the go-task (i.e., the direction of the arrow that appears on the screen) by pressing the corresponding arrow key on the keyboard. In stop-trials, the arrow is followed by a “Stop” signal after a variable stop-signal delay (FIX, fixation duration; SSD, stop signal delay; ITI, intertrial interval), instructing participants to suppress the imminent go response. The initial value of the SSD was set to 150 ms and adjusted individually and dynamically throughout the experiment (i.e., staircase procedure).

Subjects were instructed to perform a simple reaction time (RT) task, which included both Go- and Stop-trials (Lappin and Eriksen, 1966; Logan and Cowan, 1984a; Logan et al., 2014; Verbruggen et al., 2019). They started with a short practice block (32 trials) and then performed four experimental blocks. Each block was composed of a total of 128 trials, of which 96 were Go-trials (75%) and 32 Stop-trials (25%), for a total of 384 Go-trials and 128 Stop-trials. Each trial started with a black dot centered on a blank white screen for 800 ms (i.e., fixation point). In Go-trials, participants were required to respond as quickly and accurately as possible to the direction of the black arrow appearing on the screen for 100 ms (i.e., Go-stimulus). During Stop-trials, participants were asked to inhibit their motor response when two different body pictures with either a fearful or happy expression (Borgomaneri et al., 2012, 2015a,b,c, 2017, 2020b, 2021), used as Stop-signals, were presented for 70 ms, after a variable Stop-signal delay (SSD) relative to the onset of the Go-stimulus (see Figure 2).

The Stop-signal delay (SSD) between Go and Stop trials was initially set at 150 ms, but was individually and dynamically adjusted separately for each stimulus with a staircase procedure, to ensure successful inhibition in approximately 50% of the Stop-trials for each stimulus (Band et al., 2003; Matzke et al., 2018; Verbruggen et al., 2019). After each trial, the SSD value was adjusted in 5 ms steps (from a minimum of 50 ms to a maximum of 650 ms), as a function of the subject’s success or failure in stopping. Our task was designed based on the recommendations of Verbruggen et al. (2019). Finally, participants were automatically redirected to the personality traits questionnaires.^2^

### Stimuli validation

A pilot study was conducted to assess whether the images of the fearful body expression and the joyful body expression were considered equally arousing and more arousing than the neutral body expression image, and that the joyful body expression was considered the most positive among the three stimuli while the fearful body expression was considered the most negative. To this aim, 30 healthy participants (18 female; mean age ± SD: 22.6 y ± 2.7) were recruited for the stimuli validation and were not involved in the subsequent SST (i.e., main experiment). Participants were initially shown all images and had to make explicit recognition of the images based on multiple proposed alternatives. The outcome was that the images were correctly associated with the appropriate alternative. The participants were then presented with different questions to rate the stimuli’s perceived valence and arousal. The order of the different judgments was randomized across participants. Participants used an electronic five-point Likert scale ranging from one (none) to five (extremely). To investigate differences in perceived *valence* between stimuli a repeated measures ANOVA with Stimuli (Fear-Body/Joy-Body/Neutral-Body) as within-subjects factor was carried out. The analysis revealed the main effect of Stimuli [*F*_(2,58)_ = 245.02, *p* < 0.001, η_*p*_^2^ = 0.894] and Bonferroni *post hoc* comparison showed significantly higher rates (i.e., positive valence) for the joyful body expression (4.67 ± 0.55) compared to the neutral body expression (2.8 ± 0.71; *p* < 0.001; df = 58), and significantly lower rates (i.e., negative valence) for the fearful one (1.2 ± 0.41) compared to the neutral one (*p* < 0.001; df = 58). This shows that the joyful body expression was considered positive and the fearful body expression was considered negative, while the neutral one scored between these two. Similarly, to investigate differences in *arousal* among the three stimuli a repeated measures ANOVA with Stimuli (Fear-Body/Joy-Body/Neutral-Body) as within-subjects factor was carried out. The analysis again revealed the main effect of Stimuli [*F*_(2,58)_ = 12.893, *p* < 0.001, η_*p*_^2^ = 0.308] and Bonferroni *post hoc* comparison showed significantly higher rates for the negative (3.5 ± 1.31) and the positive stimuli (3.43 ± 1.01) compared to the neutral one (2.3 ± 0.99; all *p* < 0.001; df = 58). Meanwhile, no significant difference was detected between the negative and the positive stimulus (*p* = 1.00; df = 58). These results showed that the fearful, joyful, and neutral body expressions were indeed perceived as such, and that both the fearful and joyful body expression were equally more arousing than the neutral body expression.

### Data processing and analysis

Inhibitory performance on the SST was measured by computing an index of reactive inhibition, the SSRT, as already computed in a previous study (Battaglia et al., 2022a). In particular, SSRTs were estimated based on Logan and Cowan’s notion of the race-model (Logan and Cowan, 1984b). In accordance with Verbruggen et al. (2013), data were computed by adopting the integration method with the replacement of Go-omissions. More specifically, the finishing time of the Stop process was determined by integrating the go RT distribution and finding the point at which the integral is equal to the probability of responding at a given delay. The ending time of the stop process corresponded to the *n*th RT, where *n* = the number of RTs in the RT distribution of Go trials multiplied by “*p* (respond| signal).” To determine the *n*th RT, all Go trials with a response were considered, including Go-trials with a choice error and Go-trials with a premature response. Also, omissions in the Go-trials (i.e., no response before the end of the Go-trials) were assigned the maximum RT to compensate for the lack of response. Finally, premature responses in unsuccessful Stop-trials (i.e., responses executed before the Stop-signal is presented) were included in calculating the probability of responding to a delay and mean SSD. This version of the integration method produces the most reliable and least biased SSRT estimation [for further details and an exhaustive review see Verbruggen et al. (2019)].

Data were analysed offline using custom-made MATLAB scripts (The MathWorks, Inc., Natick, MA, USA) estimating SSRT as described, and all statistical analyses were performed with STATISTICA (StatSoft STATISTICA 13, Tulsa, OK, USA). Mixed-design analyses of variance (ANOVAs) were used to investigate differences within and between groups. *Post hoc* analyses were conducted with Bonferroni test and the significance threshold was set at *p* < 0.05.

## Results

### Verification of the correct assumptions underlying the stop-signal task data collected

Firstly, we verified the correct assumptions of the independent race model (Verbruggen et al., 2019). In particular, we assessed whether the mean RT on Unsuccessful Stop trials (i.e., trials in which participants could not desist from performing an action even though a Stop-signal was presented) was shorter than the mean RT on Go trials (see Table 1 for descriptive SST data). To this aim, we ran a 3 × 2 ANOVA on RTs with Trial type (Go/Unsuccessful Emotional Stop/Unsuccessful Neutral Stop) as within-subject factor and Group (Fear-Body/Joy-Body) as between-subject factor. The analysis revealed a main effect of Trial type [*F*_(2,116)_ = 105.03, *p* < 0.001, η_*p*_^2^ = 0.644]. Bonferroni *post hoc* comparisons showed RTs for Go trials were significantly longer (514.36 ms ± 16.42 ms) than both Unsuccessful Emotional Stop trials (458.40 ± 12.85 ms, *p* < 0.001; df = 116) and Unsuccessful Neutral Stop trials (459.49 ± 13.01 ms, *p* < 0.001; df = 116), while no difference emerged between those last two (*p* = 1.000). No other main effects or interaction were found to be significant (all *F* < 1.296; *p* > 0.44; η_*p*_^2^ < 0.022).

**TABLE 1:** Stop signal task (SST) behavioral data.

SST	Fear-body group		Joy-body group	
	Emotional	Neutral	Emotional	Neutral
Inhibition rate (%)	50.62 ± 5.07	50.31 ± 4.61	50.89 ± 7.12	49.95 ± 7.99
SSD (ms)	263.33 ± 98.13	259.61 ± 97.04	266.82 ± 115.63	260.99 ± 114.90
SSRT (ms)	222.30 ± 25.52	227.02 ± 26.58	237.31 ± 49.26	245.44 ± 45.81
Unsucc RT (ms)	451.04 ± 91.21	448.71 ± 90.93	465.76 ± 107.19	470.26 ± 109.79
Go RT (ms)	499.90 ± 118.94		528.53 ± 134.97	
Correct go (%)	99.11 ± 1.22		98.98 ± 1.20	

Descriptive performance of the SST is reported as means ± standard deviations. In particular, inhibition rate, stop signal delay (SSD), stop signal reaction time (SSRT), unsuccessful reaction time (Unsucc RT), go reaction time (Go RT), and correct go responses are depicted in the table for each group.

Subsequently, we ensured that the staircase procedure was successful, ascertaining that the inhibition rate (i.e., percentage of the accuracy of the stop performance when the Stop-signal is presented) was approximately 50% for all stimuli during the task (Fear-Body group: Emotional = 50.63 ± 5.07%, Neutral = 50.31 ± 4.61%; Joy-Body group: Emotional = 50.89% ± 7.12%, Neutral = 49.95% ± 7.99%). To investigate differences across groups a 2 × 2 ANOVA on the percentage of the accuracy of stop performance (i.e., inhibition rate) with Stimulus (Emotional/Neutral) as within-subject factor and Group (Fear-Body/Joy-Body) as between-subject factor was carried out. The analysis revealed that the inhibition rate did not differ between groups [*F*_(1,58)_ = 0.001, *p* = 0.97, η_*p*_^2^ < 0.001], nor was it influenced by the emotional content of the Stimulus [*F*_(1,58)_ = 3.569, *p* = 0.06, η_*p*_^2^ = 0.058]. Moreover, no Stimulus by Group interaction was found [*F*_(1,58)_ = 0.892, *p* = 0.35, η_*p*_^2^ = 0.015; see Table 1 for descriptive SST data]. These results indicated that the percentage of the accuracy of the stop performance, when the Stop-signal is presented, was comparable both for the two stimuli and for all participants regardless of the group.

Similarly, we investigated the percentage of correct responses on Go-trials across groups using a 2 × 2 ANOVA with Go-responses (Correct/Incorrect) as within-subject factor and Group (Fear-Body/Joy-Body) as between-subject factor. The analysis revealed a main effect of Go-responses [*F*_(1,58)_ = 98.161, *p* < 0.001, η_*p*_^2^ = 0.999], but no main effect of Group or Go-responses by Group interaction [*F*_(1,58)_ = 0.173, *p* = 0.68, η_*p*_^2^ = 0.003], suggesting that all participants regardless of the group, had a similar performance in discriminating the direction of the arrow presented as the Go-signal. Follow-up simple paired *t*-tests [*t*_(59)_ = 315.53, *p* < 0.001] revealed that correct Go-responses (99.04 ± 1.20%) were significantly higher than incorrect ones (0.96 ± 1.20%; see Table 1 for descriptive SST data), suggesting that the SST was correctly executed by the participants. Finally, to assess sequential effects on reaction times following Go-trials, a one-way ANOVA on the Go-RTs was performed. The analysis revealed no differences in reaction times between groups [*F*_(1,58)_ = 0.775, *p* = 0.38, η_*p*_^2^ = 0.013]; see Table 1 for descriptive SST data.

In conclusion, given these preliminary analyses, the SST data collected can be considered reliable and the assumption of correct inhibition rate has been verified. Thus, it is possible to reliably estimate the SSRT values (Verbruggen et al., 2019).

### Valence-independent emotional content of stimuli enhances the ability to disrupt an ongoing action

**FIGURE 3:**
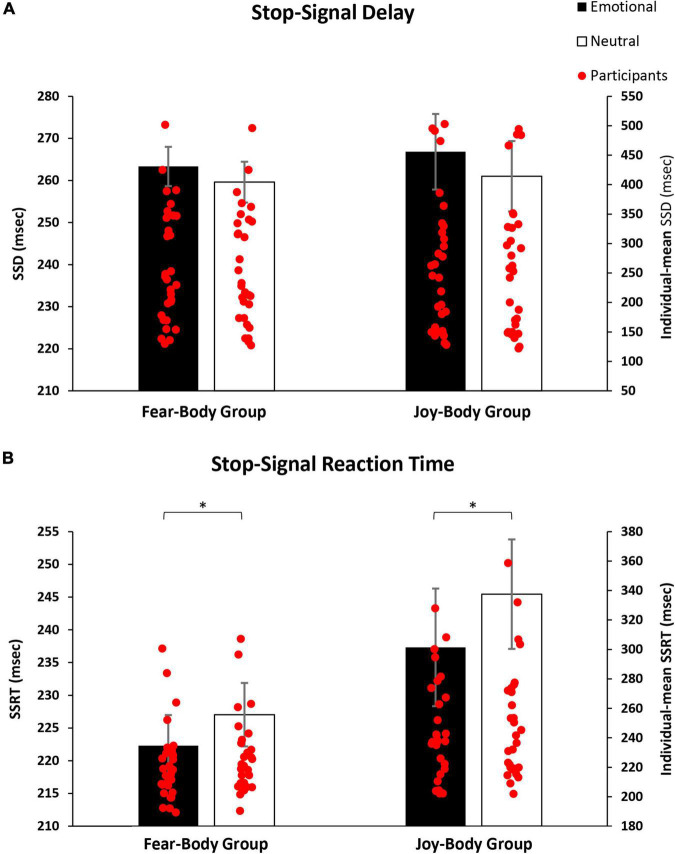
Bar graphs of the experimental results. In panel **(A)**, the graph shows the mean stop-signal delay (SSD), demonstrating that the emotional content of stimuli influenced the participant’s action execution leading to a specific differentiation of SSD, given the successful staircase procedure. In panel **(B)**, the graph shows the mean stop-signal reaction time (SSRT), demonstrating that participants showed a better inhibitory process when facing emotive Stop-signals as compared to neutral ones, regardless of the group. *Indicate significant comparisons (*p* < 0.05), and error bars represent S.E.M.

Prior to the main analysis of the study, SSD data were analysed using a 2 × 2 ANOVA with Stimulus (Emotional/Neutral) as within-subject factor and Group (Fear-Body/Joy-Body) as between-subject factor. The analysis revealed only the main effect of Stimulus [*F*_(1,58)_ = 9.995, *p* = 0.002, η_*p*_^2^ = 0.147] and Bonferroni *post hoc* comparison showed significantly longer SSD for emotional (265.08 ± 106.34 ms) than neutral Stop-signals stimuli (260.30 ± 105.44 ms; *p* = 0.002; df = 58). Furthermore, no main effect of Group [*F*_(1,58)_ = 0.008, *p* = 0.93, η_*p*_^2^ < 0.001] and no effect of Group by Stimulus interaction were found to be significant [*F*_(1,58)_ = 0.487, *p* = 0.49, η_*p*_^2^ = 0.008; see Table 1 for descriptive SST data]. As expected, the emotional content of the stimuli influenced the participant’s actions execution, leading to a specific differentiation of SSD that was properly adjusted given the successful staircase procedures (see Figure 3A). Crucially, to verify the main hypothesis of the present study, SSRT data were analysed using a 2 × 2 ANOVA with Stimulus (Emotional/Neutral) as within-subject factor and Group (Fear-Body/Joy-Body) as between-subject factor. Results showed the main effect only of Stimulus [*F*_(1,58)_ = 10.808, *p* = 0.002, η_*p*_^2^ = 0.157]. Bonferroni *post hoc* comparisons showed that SSRTs were significantly lower (*p* = 0.002; df = 58) for emotional stimuli (229.81 ± 39.62 ms) with respect to neutral ones (236.23 ± 38.28 ms). No other main effects or interaction were found to be significant (all *F* < 2.966; *p* > 0.09; η_*p*_^2^ < 0.049; see Table 1 for descriptive SST data). To further investigate the effect of emotion in the SSRT, follow-up simple paired *t*-tests revealed that SSRT was significantly reduced for the negative emotion condition compared to its neutral counterpart in the Fear-Body group [*t*_(29)_ = -2.63, *p* = 0.01] and the positive emotion condition compared to its neutral counterpart in the Joy-Body group [*t*_(29)_ = -2.34, *p* = 0.03; see Figure 3B]. Finally, these results showed that participants were more capable in inhibiting responses with emotional Stop signals compared to neutral ones, irrespective of their valence.

Finally, to explore the relations between the better action inhibition when facing emotional stimuli and personality traits, a regression analysis was performed. An index representing the inhibition for emotional stimuli (i.e., SSRT of the emotional stimuli minus the SSRT of the neutral stimulus) was considered as the dependent variable in a stepwise regression model, whereas scores for the STAI-Y2 and BIS11 subscales were entered as predictors. The regression model was not found to be significant [*R*^2^ = 0.070; *F*_(4,55)_ = 1.044; *p* = 0.39], indicating that personality traits do not impact on participants’ inhibitory performance.

## Discussion

Positive emotional expressions have been found to capture attention automatically (Miyazawa and Iwasaki, 2010; Gupta et al., 2016; Torrence et al., 2017) as well as negative stimuli. Indeed, the activation of visual areas occurs rapidly when viewing emotional bodies, as shown by early components of event-related potentials (ERPs), such as the P1, N1, and N190 (van Heijnsbergen et al., 2007; Jessen and Kotz, 2011; Borhani et al., 2015), suggesting a rapid allocation of cognitive resources for monitoring biologically relevant signals. A growing number of preclinical evidence have also revealed the interconnection and interference of those behavioral domains (Telegdy et al., 2010, 2011; Tanaka et al., 2011; Palotai et al., 2014) that may eventually lead to development of mental illnesses (Martos et al., 2022; Tanaka and Vécsei, 2022; Tanaka et al., 2022a,b). Moreover, emotional bodies have been found to early modulate the corticospinal excitability of an observer (Borgomaneri et al., 2015a,b,c, 2017, 2020b, 2021), highlighting the motor system’s involvement during perception of emotional bodies. However, it is unclear whether positive expressions have the same ability as negative stimuli to boost our action control. Here, we used an SST with an emotional negative or positive body posture as stop signal, in which the emotional stimuli were task-irrelevant and we found that both types of emotional arousal-matched stimuli were able to ameliorate our action control (i.e., reducing the SSRT with respect to the neutral body posture). These results are in line with our previous findings (Battaglia et al., 2022a) which demonstrated that different kinds of negative stimuli (i.e., facial expressions, body postures and the SARS-CoV-2 image) are equally able to ameliorate action suppression. Together with the present findings, our findings support the “Dual competition framework” (Pessoa, 2009), which proposed that the potency of the emotional stimuli is able to modulate their influence on executive functions. Namely, when the emotional content of the stimuli is low in threat (or positive), the processing is biased in favor of the emotional item, thus emotional stimuli would enhance the response inhibition, because they require fewer resources to process, thus leaving enough resources required for response inhibition, while when the emotional content of the stimuli is high in threat, it recruits a “common-pool resources” of executive functions, impairing them. This theory suggests that with respect to neutral stimuli, emotional low-level images generated enhanced sensory representations of the stop stimulus in the visual cortex (Pessoa et al., 2002; Pourtois et al., 2004; Kolassa and Miltner, 2006; Smith et al., 2013), leading to a stronger representation of the stop signal and consequently enhanced stopping performance. Our results are also in line with those reported by Pessoa et al. (2012), in which task-irrelevant fearful and happy facial expressions were found to increase action control compared to neutral stimuli. Here, we not only demonstrated that other kinds of stimuli (body postures versus faces) are able to produce similar effects, but we also employed arousal-matched stimuli in order to ensure that the difference between emotional and neutral stimuli in response inhibition would be completely attributed to arousal, while no influence of valence was found.

Interestingly, our data are in contrast with the recent findings of Gupta and Singh (2021), who reported better action control when facing negative facial expressions as stop signals, while no differences were found between positive and neutral stimuli. However, in their task-irrelevant SST, the authors employed angry facial expressions rather than fearful stimuli as in the present study and that of Pessoa. Gupta and colleagues suggested that, compared to fearful and happy stimuli, angry stimuli would facilitate avoidance related behavior, being perceived as aversive more in line with the “Approach and avoidance framework” (Marsh et al., 2005; Hammer and Marsh, 2015). On the other hand, a previous study employing angry facial expressions found the opposite results, namely longer SSRTs when angry facial stimuli were presented, although in this case the facial stimulus was the go-signal requiring a gender discrimination task (Rebetez et al., 2015). Moreover, Williams et al. (2020) found better action control only in older adults and when emotions were task-relevant, as in the findings of Nayak et al. (2019). In line with these results, in a series of go-nogo studies, Mirabella and colleagues showed that emotional facial expressions affect motor control only when task relevant, i.e., when participants needed to pay attention to the emotional content of the stimuli valence to give a correct response (Mirabella, 2018; Mancini et al., 2020, 2022; Mirabella et al., 2022). Therefore, it seems that several variables determine the results, such as whether the emotional stimuli have a beneficial or a detrimental effect and whether positive and negative emotions have a similar effect on action control. The evidence reported here suggests that important factors need to be considered, first, including the selected negative emotion that is paired with the happy/positive stimuli (i.e., angry, fearful or disgusted), then that the relevance of the emotional stimuli in the SST, and thirdly, whether the emotional stimuli are used as go or as stop stimuli [see Battaglia et al. (2021) for a deeper discussion]. A potential limitation of our study is the use of a between-subjects design, which prevents a direct comparison between the performance facing happy and fearful body postures. Moreover, future studies should investigate whether another domain of inhibitory control, as proactive inhibitory control (i.e., the ability to adapt the motor strategy according to the current context) (Aron, 2011), is affected by the emotional content of the stimuli, as we have shown for reactive inhibition. Finally, future studies may consider to use social and/or biological stimuli as go-signals to test their ability to impact on action control due to their capability to capture attention more than non-biological signals (Battaglia et al., 2022b).

Learning how emotional information impacts action control, and thus inhibitory processes, is highly expedient to understand the neural mechanisms underlying the deficient inhibitory control, which is crucially affected across different psychopathologies and mood disorders, such as anxiety, bipolar disorder, obsessive-compulsive disorder, schizophrenia, and autism [for a review, see Battaglia et al. (2021)]. Future studies could usefully delve more deeply into the neural bases of the interaction between emotion and action control [i.e., implementing the NIBS in an SST with emotional stimuli (Borgomaneri et al., 2020a)], both in healthy participants as well as in the clinical population.

## Conclusion

Here, we have demonstrated that task-irrelevant emotional arousal-matched happy and fearful body postures are able to ameliorate our reactive action inhibition when presented as stop signals in an SST task, in line with the “dual competition framework” (Pessoa, 2009). Here, we have discussed several factors that may have produced different results in the literature. Future studies could systematically investigate the manipulations of such factors to strengthen the results of the present study, which will further help examine the role of valence in response inhibition.

## Data availability statement

The datasets collected and analyzed during this current study are available from the corresponding authors on reasonable request, due to concerns about privacy, health status (i.e., SARS-CoV-2 diagnosis), and confidentiality of our participants.

## Ethics statement

The studies involving human participants were reviewed and approved by the University of Bologna. The patients/participants provided their written informed consent to participate in this study.

## Author contributions

SBa and SBo conceived and designed the study and wrote the manuscript. PC developed the local-only version of a classical Stop-Signal Task in JavaScript, while SBa developed the jsPsych-version of the Stop-Signal Task customized for the present study. CDF and CN performed the SST data collection, questionnaire scoring, and analysis. SBa performed the SST data analysis and designed the figures and table. All authors approved the final version of the manuscript for submission.

## Footnotes

1
https://www.ecma-international.org/publications/standards/Ecma-006.htm

2
https://www.google.com/forms/about/
